# Burning pain on the upper labial mucosa

**DOI:** 10.1016/j.jdcr.2023.08.012

**Published:** 2023-08-23

**Authors:** Christopher J. Fay, Jacqueline D. Lippert, Nicole R. LeBoeuf

**Affiliations:** a Department of Dermatology, Brigham and Women’s Hospital, and the Center for Cutaneous Oncology, Dana-Farber/Brigham Cancer Center, Boston, Massachusetts; b Department of Internal Medicine, Atrium Health Wake Forest Baptist Medical Center, Winston-Salem, North Carolina; c Harvard Medical School, Boston, Massachusetts

**Keywords:** geographic lip, geographic tongue, psoriasis, streptococcal pharyngitis

## Case description

**Fig 1:**
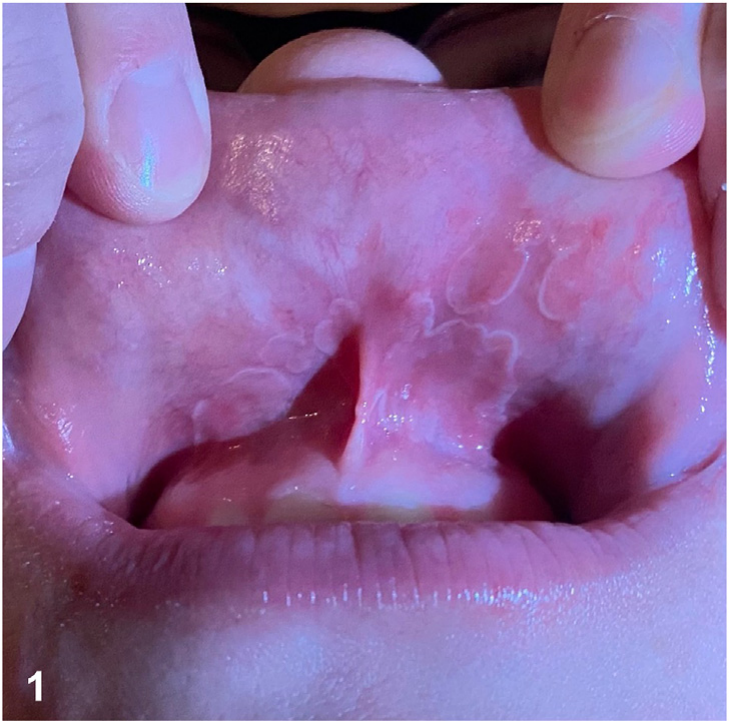

A 36-year-old pregnant woman presented with 1 week of burning pain on the upper labial mucosa and new onset headache. Six days prior, the patient’s 3-year-old child tested positive for streptococcal pharyngitis after exposure at daycare and was treated with antibiotics. Physical examination was remarkable for pink thin plaques with white, polycyclic borders on the upper labial mucosa (Fig 1) that could not be wiped away. The patient was empirically treated for streptococcal pharyngitis with penicillin V owing to her symptoms and pregnancy status. Lip pain and rash began to improve within 3 days and resolved completely within 4 weeks.

**Question 1: What is the most likely diagnosis?**

A. Lichen planus
B. Candidiasis
C. Aphthous stomatitis
D. Geographic lip
E. Herpetic gingivostomatitis

**Answers:**

A. Lichen planus – Incorrect. Oral lichen planus may involve labial mucosa and can be associated with sensitivity to acidic or spicy food similar to geographic tongue. However, this condition presents more commonly with bilateral white reticulations, which are frequently associated with erythema and erosions and yellow fibrinous exudate.
B. Candidiasis – Incorrect. Although oral candidiasis may present with burning pain and can involve any mucosal surface, this condition classically presents as curd-like white papules and plaques that can be wiped off.
C. Aphthous stomatitis – Incorrect. This common condition affects approximately 20% to 50% of the population and favors labial mucosa but presents more commonly with ovoid ulcers with a white-to-yellow base and an erythematous rim.
D. Geographic lip – Correct. Geographic tongue is a benign, inflammatory disorder of unclear etiology that predominantly involves the dorsal and lateral tongue. Ectopic geographic tongue (in this case, geographic lip specifically) may involve any oral mucosa and classically presents as flattened or atrophic lesions varying from a few millimeters to several centimeters with a serpiginous white border.
E. Herpetic gingivostomatitis – Incorrect. Herpetic gingivostomatitis may involve the labial mucosa but frequently presents with vesicles at the vermillion border that break to form erosions and ulcers that subsequently crust.

**Question 2: Geographic tongue shares strong similarities with what common dermatologic condition?**

A. Psoriasis
B. Erythema multiforme
C. Pityriasis rosea
D. Discoid lupus erythematosus
E. Rosacea

**Answers:**

A. Psoriasis – Correct. Current literature suggests clinical, histologic, and immunohistochemical similarities between geographic tongue and psoriasis.^1^^,^^2^ In fact, some authors have argued that geographic tongue may represent oral manifestations of psoriasis, although this remains controversial. Moreover, a systemic review and meta-analysis indicated an association between geographic tongue and psoriasis (odds ratio: 3.53; 95% CI: [2.56-4.86]; *P* = .03).^1^ Interestingly, this patient had a streptococcal exposure, new onset headache, and was treated empirically for streptococcal pharyngitis at the time of lip symptoms. There is a well-established association between acute guttate psoriasis and preceding streptococcal infection.^3^ If ectopic geographic tongue shares similarities to the immune-mediated pathogenesis of psoriasis, it raises the possibility that the lesions on the patient’s upper labial mucosa occurred secondary to streptococcal infection.
B. Erythema multiforme – Incorrect. There is no clear relationship between erythema multiforme and geographic tongue.
C. Pityriasis rosea – Incorrect. There is no clear relationship between pityriasis rosea and geographic tongue.
D. Discoid lupus erythematosus – Incorrect. There is no clear relationship between discoid lupus erythematosus and geographic tongue.
E. Rosacea – Incorrect. There is no clear relationship between rosacea and geographic tongue.

**Question 3: Which of the following statements is FALSE?**

A. Migratory stomatitis is another, appropriate name for geographic lip.
B. Ectopic geographic tongue was first described in 1955.
C. Spicy or acidic foods may exacerbate symptoms of geographic tongue, both lingual and ectopic.
D. Although the diagnosis of ectopic geographic tongue is clinical only, biopsies may demonstrate elongation of rete edges, parakeratosis, Munro abscesses, and suprapapillary thinning.
E. Geographic tongue affects approximately 1 in 5 individuals.

**Answers:**

A. Migratory stomatitis is another, appropriate name for geographic lip – Incorrect. This is true. There are many names that have been used for ectopic geographic tongue, such as migratory stomatitis, stomatitis areata migrans, erythema circinata migrans, annulus migrans, and geographic stomatitis.
B. Ectopic geographic tongue was first described in 1955 – Incorrect. This is true. This phenomena was first described in a 30-year-old Maltese man in 1955.^4^
C. Spicy or acidic foods may exacerbate symptoms of geographic tongue, both lingual and ectopic – Incorrect. This is true. Although some patients may be asymptomatic, burning pain is often related to eating spicy or acidic foods.
D. Although the diagnosis of ectopic geographic tongue is clinical only, biopsies may demonstrate elongation of rete edges, parakeratosis, Munro abscesses, and suprapapillary thinning – Incorrect. This is true. Similar to psoriasis, biopsies of these lesions may demonstrate elongation of rete edges, parakeratosis, Munro abscesses, and suprapapillary thinning.^2^
E. Geographic tongue affects approximately 1 in 5 individuals – Correct. This statement is false. The prevalence of geographic tongue is 1% to 4.8%, and ectopic geographic tongue is rarely described.^5^

## Conflicts of interest

The authors declare no conflicts of interest with respect to the research, authorship, and/or publication of this article. Nicole R. LeBoeuf is a consultant and has received honoraria from Bayer, Seattle Genetics, Sanofi, Silverback, Fortress Biotech, and Synox Therapeutics outside the scope of the submitted work.
